# Social capital and healthy eating among two ethnic minority groups in Yunnan Province, Southwest China: the mediating role of social support and nutrition knowledge

**DOI:** 10.3389/fnut.2024.1273851

**Published:** 2024-05-31

**Authors:** Qiang Zhang, Chunrong Huangfu, Qingqing Wan, Weiwei Su, Xiao Zhu, Bin Yu, Xiangdong Min, Zhitao Liu

**Affiliations:** ^1^Department of Nutrition and Food Hygiene, Yunnan Center for Disease Control and Prevention, Kunming, China; ^2^Department of Pediatrics, The First People’s Hospital of Yunnan Province, Kunming, China; ^3^Department of Epidemiology, School of Public Health, Wuhan University, Wuhan, China

**Keywords:** social capital, healthy eating, structural equation model, mediation, nutrition knowledge, social support

## Abstract

**Background:**

Although social capital has been linked to dietary intake particularly in disadvantaged populations, little is known about the mechanisms. This study aimed to investigate whether social support (SS) and nutrition knowledge (NK) mediate the association between social capital and healthy eating habits.

**Methods:**

A probability sample of two ethnic minority groups in Yunnan Province, Southwest China were included (*n* = 1,033, mean age 47.5 ± 14.7 years). Bonding and bridging social capital (BOC and BRC) were assessed with the Personal Social Capital Scale (PSCS-16). Dietary data were evaluated with the Chinese Healthy Eating Index (CHEI), a measure of diet quality which reflects adherence to the Chinese Dietary Guidelines. NK and SS were measured with a validated questionnaire and scale, respectively. Structural Equation Modeling was used to calculate the direct, indirect and total effects of social capital on CHEI scores.

**Results:**

The mean score of CHEI was 57.4 ± 9.8, which was significantly lower in men and older people. Low adherence to dietary guidelines were to observed in the consumption of dairy, beans, nuts, animal-source food vegetables and fruits. BOC and BRC were positively associated with CHEI score (β = 0.37 and 0.38, all *p* < 0.05). Social support and nutrition knowledge mediated 45.9 and 39.5% of the total effect of social capital on CHEI score, respectively.

**Conclusion:**

Social capital appears to enhance adherence to dietary guidelines by improving nutrition knowledge and social support. Nutrition promotion programs therefore should consider incorporating strategies that foster social capital development, particularly in disadvantaged populations.

## Introduction

It is estimated that over 20% of global adult deaths each year can be attributed to dietary risk factors, such as excessive salt intake, and inadequate consumption of vegetables, fruits, and whole grains (1). Current theoretical frameworks like the socio-ecological model, holds that dietary habits are shaped by individuals’ interactions with their social and physical surroundings (2). Social capital, as resources embedded in social networks, has been found to be favorable for healthy eating in various cultural contexts and populations (3–5). Therefore, further research on how social capital affects diet intake is crucial to inform policy decision.

Social capital is recognized as an important health determinant, probably due to the positive impacts of social support and information gained in social networks (6). A national representative study conducted in the United States has shown that individuals with higher social capital are more likely to acquire health information from health professionals (7). Health information, particularly in the realm of nutrition often predicts better adherence to healthy dietary patterns like Mediterranean diet (8, 9). Similarly, another study in China found that older adults with extensive social networks and active social participation tend to have higher health literacy (defined as the motivation and ability of individuals to gain access, understand and use information in ways which promote and maintain good health) and follow healthier diets (10). On the other hand, social support is also essential for the adoption of healthy lifestyle behaviors. People with higher social capital often receiving better support from their social environments. Numerous studies have highlighted the significant role of support from families, friends and peers in influencing personal dietary intake (11). A study in Canada indicates that social support even has a stronger influence on healthy eating than physical environment (12). These findings suggest that social support and nutrition knowledge may act as mediators between social capital and diet quality, although further research is needed to determine if these relationships hold true in the Chinese context.

People would acquire various types of social capital through different social connections. Putnam posits two distinct social capital, bonding and bridging (13). The former refers to interactions between homogeneous members of a community such as families, close friends and neighbors, while the later refers to interactions between heterogeneous members connecting together through various social organizations (14). Distinguishing these types of social capital is crucial in nutrition research because they can have varying effects on dietary intake (15). For instance, a study conducted in India found that bridging social capital, expressed as household connections with development-oriented community-based organizations, was positively associated with child nutrition, while bonding capital had the opposite effect (16). This disparity may be attributed to the fact that while bonding social capital can offer substantial support, its “inward” nature tends to reinforce traditional norms and habits (17). In contrast, the “outward” bridging social capital could introduce more novel information and ideas (18). Therefore, the relationship between bonding social capital and dietary intake would be different from that of bridging social capital. However, there is a scarcity of relevant studies on this subject.

Yunnan is a multi-ethnic, less-developed province locates in Southwest China. With rapid economic growth in recent years, dietary imbalance has become prevalent in ethnic minority groups (19, 20). Previous research indicates that social capital has a greater impact on socioeconomically disadvantaged populations (21, 22). Therefore, the purpose of this study is to investigate the association between social capital and dietary guidelines compliance among ethnic minorities, as well as the mediating effects of social support and nutrition knowledge in this relationship.

## Materials and methods

### Study design, setting and participants

Yunnan province boasts the largest number of ethnic groups in China. Some of these ethnic groups, such as Bu Lang and Pu Mi, have relatively small populations and only reside in specific regions within Yunnan. The study sites were selected based on the towns with the highest population of Bu Lang or Pu Mi ethnicity. Two villages were then randomly chosen from each town using the Probability Proportional to Size method. Subsequently, 150 households were randomly selected from each village using local household registration information. All adults in these households were invited to participate in the study, excluding individuals on prescribed diets or those with serious physical or mental illnesses. Data were collected through a face-to-face interview conducted by trained local health workers.

We aimed at recruiting 550 participants from each ethnicity. The sample size was determined through rigorous power analysis, and further details on sample size calculation have been described elsewhere (23). After excluding individuals with missing data on socio-demographics data (*n* = 13), reporting implausible energy intakes (< 800 kcal per day or > 6,000 kcal for men; and < 600 kcal or > 4,000 kcal for women, *n* = 44), or following prescribed diets (*n* = 9), a total of 1,033 subjects were included in the final analyses.

### Measurements

#### Social capital (predictor)

Social capital was assessed using the Personal Social Capital Scale-16 (PSCS-16), comprising 16 items divided eight measuring bonding capital (BOC) and eight items measuring bridging capital (BRC) (24). BOC evaluates a person’s connections with various individuals, including family members, relatives, friends, classmates and colleagues, while BRC assesses connections with different types of social organizations, including political/economic and recreational/cultural organizations. Both BOC and BRC were evaluated based on network size, trust, resources and reciprocity. Responses for each item were ranked on a 5-point Likert scale (1 = a few, 2 = less than average, 3 = average, 4 = more than average, 5 = a lot). The total score of BOC and BRC range from 8 to 40, with higher scores indicating greater social capital. Previous studies in China and abroad has demonstrated reliability and validity of this scale (25, 26).

#### Nutrition knowledge (mediator)

Nutrition knowledge was assessed using a questionnaire from the China Health and Nutrition Survey (CHNS), which includes 17 diet-related statements (27). Responses for each statement were ranked on a 5-point Likert scale (strongly disagree = 1, disagree = 2, neutral = 3, agree = 4 and strongly agree = 5). Correct response for each item received 1 point, while incorrect response receives 0 point. The total score was calculated by summing up the scores for all the 17 statements, resulting in a range of 0 to 17. Higher total scores indicated a greater level of nutrition knowledge.

#### Social support (mediator)

Social support was assessed using the Multi-dimensional Scale of Perceived Social Support (MSPSS), a widely utilized tool that has been adapted for use in various countries (28, 29). The MSPSS comprises 12 items that evaluate support from family, friends and significant others. Response for each item was ranked on a 5-point Likert scale, ranging from strongly disagree (1) to strongly agree (5). The total score was calculated by summing scores of the 12 items, resulting a range of 12 to 60. A higher total score indicates higher levels of perceived social support.

#### Dietary data collection

Dietary data were collected using a 100-item food frequency questionnaire (FFQ), a lifestyle questionnaire, and a household condiment inventory. Participants were asked to recall the frequency of consumption and estimated portion size for each food item over the previous 12 months. The lifestyle survey included questions about drinking frequency and the average quantity of alcoholic beverages consumed over the past 12 months. The household condiment inventory recorded family size and the consumption of salt and cooking oils over the previous month. Daily intake of food items, alcohol, cooking oils, and salt were calculated based on these data.

Energy intake was calculated using the daily dietary data in conjunction with the China Food Composition Table (30). Dietary data was then categorized into food groups according to with the Dietary Guidelines for Chinese 2016 (DGC-2016) for further analysis.

#### Chinese healthy eating index (outcome)

Dietary assessment conducted using the Chinese Healthy Eating Index (CHEI), a validated and reliable measure of diet quality as per DGC-2016. The CHEI comprises 17 components, including total grains, whole grains and mixed beans, tubers, total vegetables, dark vegetables, fruits, dairy, soybeans, fish and seafood, poultry, eggs, seeds and nuts, red meat, cooking oils, salt, added sugars and alcohol. Scoring for these components is based on energy density (as amounts per 1,000 calories of intake). According to recommendations of DGC-2016, fruits, cooking oils and salt are assigned 10 points each, while the remaining 14 components are assigned 5 points each. The total CHEI score is calculated by summing the scores of all components, with the potential range of CHEI scores being between 0 and 100. A higher CHEI score indicates greater adherence to the Dietary Guidelines for Chinese and higher overall diet quality. CHEI components and standard for scoring can be found in Supplementary Table S1.

#### Covariates

Covariates such as age, sex, ethnicity, education and income level were considered based on findings from a previous study in the population (31). Age was categorized into four groups (18–34, 35–44, 45–59, and ≥ 60 years) in descriptive statistics, while treated as a continuous variable in the modeling analyses. Sex and ethnicity were treated as binary variables (1 = men, 2 = women; 1 = Bu Lang, 2 = A Chang). Educational attainment was classified into three levels (primary school or less, middle school, high school or more). Household income *per capita* over the previous year was used to measure income, which was then divided into three groups (<5,000 Yuan, 5,000–9,999 Yuan and ≥ 10,000 Yuan).

### Statistical analysis

Descriptive statistics (percentage, mean and standard deviation, SD) were used to characterize the study population. One-way ANOVA and *t*-test were used to compare the differences of BOC, BRC, NK, MSPSS and CHEI scores across sociodemographic variables. The relationships among these variables were examined in a sequential manner. Firstly, confirmatory factor analyses (CFA) were performed to assess the psychometric properties of the PSCS-16 and MSPSS. Secondly, Pearson correlation coefficients were computed to evaluate the structural associations among social capital, nutrition knowledge, social support and CHEI scores. Finally, a structural equation modeling approach was used to examine the direct and indirect relationships between social capital and CHEI, while controlling for age, sex, ethnicity, education, and income levels. Bootstrapping was used to test the significance of the indirect effects. Based on recommendations (32), Model fit was evaluated using various indices and criteria, including the ratio of chi-square to degrees of freedom (χ^2^/df ≤ 5), Root Mean Square Error of Approximation (RMSEA ≤0.08), Comparative Fit Index (CFI ≥ 0.90), and Tucker-Lewis Index (TLI ≥ 0.90). Data analysis was performed using IBM SPSS Statistics 20.0 and AMOS 23.0 software. A *p* value of less than 0.05 was considered statistically significant.

## Results

### Descriptive statistics

Table 1 presents an overview about the sociodemographic characteristics and model variables of the study population. The total sample consisted of 465 men and 568 women, with a mean age of 47.5 years. Bu Lang and Pu Mi ethnicity accounted for approximately 50% of the sample, respectively. Nearly two-thirds of the subjects had an annual income less than 10,000 RMB Yuan (equivalent to 1,500 US dollars), and 46.8% had only primary school education or no formal education. The mean values of BOC, BRC, NK, MSPSS and CHEI scores were 22.4, 21.3, 7.6, 35.5 and 57.4, respectively, and most of the variables varied across the sociodemographic groups.

**Table 1:** Sociodemographic characteristics and model variables of the study population (*n* = 1,033).

	*n* (%)	Mean ± SD				
		BOC	BRC	NK	SS	CHEI
Total	1,033	22.4 ± 3.2	21.3 ± 2.9	7.6 ± 2.0	35.5 ± 3.5	57.4 ± 9.8
Ethnicity						
Bu Lang	511 (49.5)	22.0 ± 3.5	21.1 ± 2.9	7.3 ± 2.0	35.3 ± 3.7	57.0 ± 9.0
Pu Mi	522 (50.5)	22.7 ± 2.9	21.5 ± 2.8	7.8 ± 1.9	35.8 ± 3.2	57.8 ± 9.9
*p*-value		<0.01^**^	0.01^*^	<0.01^**^	0.02^*^	0.16
Sex						
Men	465 (45.0)	22.2 ± 3.0	20.9 ± 2.8	7.3 ± 1.9	34.9 ± 3.5	54.9 ± 8.9
Women	568 (55.0)	22.6 ± 3.4	21.6 ± 2.9	7.8 ± 2.0	36.1 ± 3.3	59.3 ± 9.9
*p*-value		<0.05^*^	<0.01^**^	<0.01^**^	<0.01^**^	<0.01^**^
Mean age (years)	47.5					
Age group (years)						
18–34	228 (22.1)	23.3 ± 3.5	21.9 ± 2.7	7.9 ± 1.9	35.7 ± 3.8	59.5 ± 8.5
35–44	224 (22.7)	22.8 ± 3.0	21.4 ± 2.8	7.8 ± 1.9	35.5 ± 3.3	58.0 ± 9.9
45–59	368 (35.6)	22.1 ± 3.2	21.1 ± 3.0	7.5 ± 1.9	35.6 ± 3.2	56.8 ± 9.7
≥60	213 (20.6)	21.6 ± 3.0	20.9 ± 2.6	7.2 ± 2.0	35.4 ± 3.5	55.5 ± 9.9
*p*-value		<0.01^**^	<0.01^**^	<0.01^**^	0.85	<0.01^**^
Education						
Primary school or less	483 (46.8)	21.8 ± 3.3	20.9 ± 2.8	7.2 ± 2.1	35.5 ± 3.2	57.3 ± 9.6
Middle school	313 (30.3)	22.5 ± 3.0	21.3 ± 2.8	7.7 ± 1.7	36.0 ± 3.3	57.0 ± 9.9
High school or more	237 (22.9)	23.5 ± 3.0	21.9 ± 2.9	8.1 ± 1.9	35.0 ± 4.1	58.2 ± 9.6
*p*-value		<0.01^**^	<0.01^**^	<0.01^**^	<0.01^**^	0.33
Income (Yuan per year)						
<5,000	275 (26.6)	22.3 ± 2.9	21.1 ± 3.1	7.7 ± 2.0	35.7 ± 3.3	57.5 ± 9.9
5,000–9,999	388 (37.6)	22.1 ± 3.0	21.1 ± 2.7	7.4 ± 1.9	35.2 ± 3.3	57.2 ± 9.5
≥10,000	370 (35.8)	22.7 ± 3.6	21.6 ± 2.9	7.7 ± 2.0	35.8 ± 3.7	58.6 ± 9.4
*p*-value		0.03^*^	0.02^*^	0.08	0.09	0.16

^*^ *p* < 0.05, ^**^ *p* < 0.01.

Table 2 shows the component scores and total scores of the CHEI. The total CHEI score was 57.4, corresponding to a score rate of 57.4%. Based on the score rate, the adherence to dietary guidelines for each component from high to low as follows: added sugar (94.0%), alcohol (84.0%), cooking oils and salt (77.5%), grains and tubers (54.7%), vegetables and fruits (48.5%), animal-source foods (47.5%) and dairy, beans and nuts (37.3%).

**Table 2:** The component scores of the CHEI (*n* = 1,033).

CHEI component	Score range	Mean score	Score rate (%)
Grains and tubers	0–15	8.2 ± 3.5	54.7
Vegetables and fruits	0–20	9.7 ± 4.2	48.5
Animal-source foods	0–20	9.5 ± 4.1	47.5
Dairy, beans and nuts	0–15	5.6 ± 3.3	37.3
Cooking oils and salt	0–20	15.5 ± 4.3	77.5
Added sugar	0–5	4.7 ± 0.3	94.0
Alcohol	0–5	4.2 ± 1.5	84.0
Total score	0–100	57.4 ± 9.8	57.4

### Confirmatory factor analyses

The results of CFA indicated that the two-factor structure of the PSCS-16 fit the data well (χ^2^/df = 1.81, *p* = 0.02; CFI = 0.96; RMSEA = 0.03; TLI = 0.98). Because all possible coefficients were estimated, the three-factor model of MSPSS was saturated (CFI = 1.00, RMSEA = 0.00), and the high loading factors implied that support from families, friends and significant others were all indicators of social support. Results of confirmatory factor analyses can be found in Supplementary Figures S1, S2.

### Correlations between the model variables

Table 3 displays the correlations among social capital, nutrition knowledge, social support and CHEI scores. The results indicated that bonding and bridging social capital showed positive associations with nutrition knowledge, perceived social support and CHEI scores. Additionally, nutrition knowledge and perceived social support exhibited positive associations with CHEI scores.

**Table 3:** Correlations between SC, SS, NK, and CHEI (*n* = 1,033).

	BOC	BRC	NK	SS	CHEI
BOC	1.00	0.45^**^	0.47^**^	0.45^**^	0.51^**^
BRC		1.00	0.51^**^	0.40^**^	0.52^**^
NK			1.00	0.29^**^	0.60^**^
SS				1.00	0.51^**^
CHEI					1.00

^*^ *p* < 0.05, ^**^ *p* < 0.01.

### SEM for the overall diet quality

**Figure 1:**
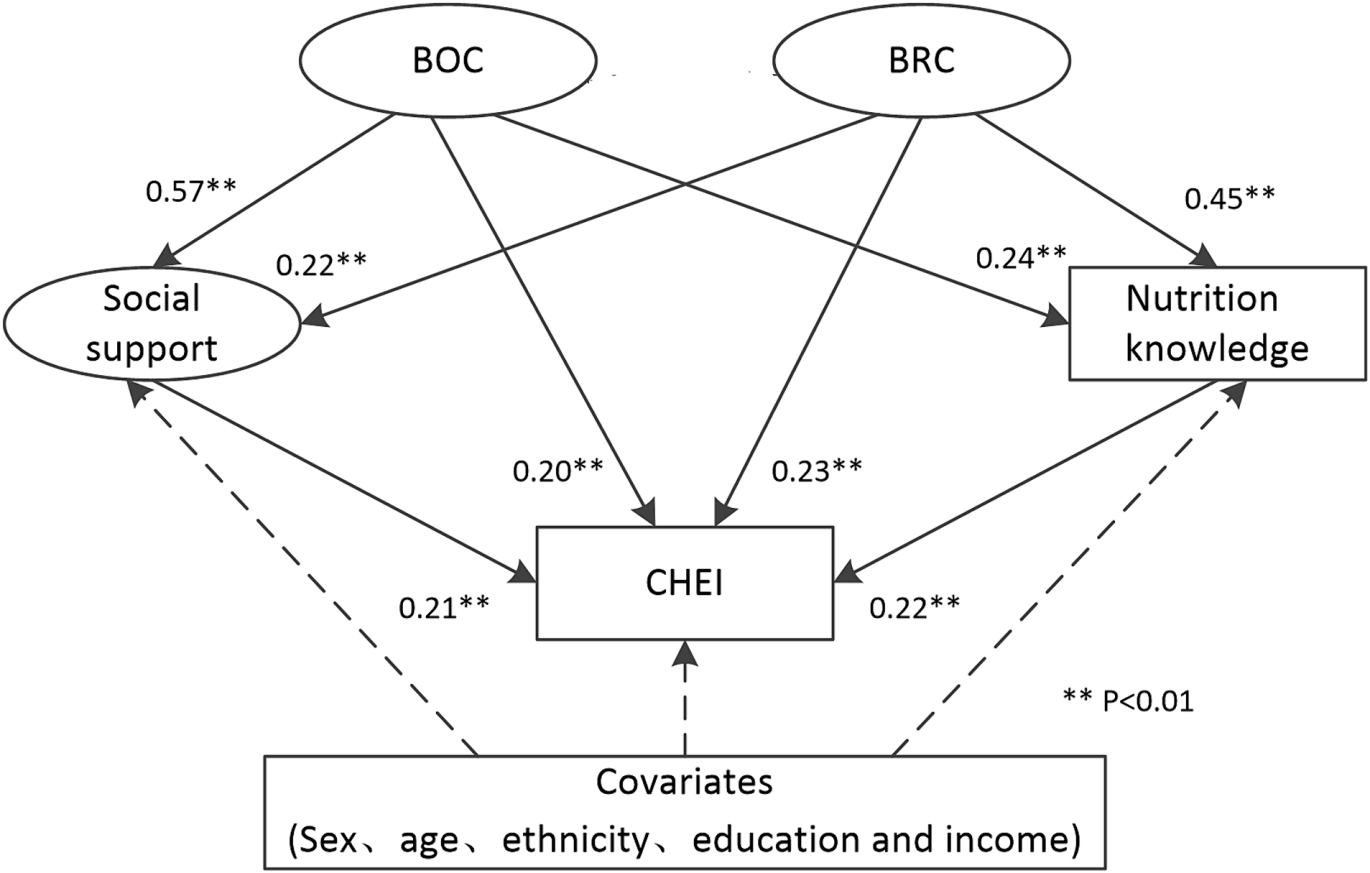
Direct and indirect pathways from social capital to CHEI scores.

The framework and results are presented in Figure 1. The model demonstrated a satisfactory fit with CHEI score as the dependent variable (*χ^2^/df* = 3.65, *p* < 0.01; CFI = 0.93; RMSEA = 0.05; TLI = 0.91). Bonding social capital were associated with social support, nutrition knowledge and CHEI scores (β = 0.57, 0.24 and 0.20, respectively, all *p* < 0.01). Similarly, bridging social capital were associated with social support, nutrition knowledge and CHEI scores (β = 0.22, 0.45 and 0.23, respectively, all *p* < 0.01). Social support and nutrition knowledge were also found to be associated with CHEI scores (β = 0.21 and 0.22, respectively, all *p* < 0.01).

Table 4 summarizes direct, indirect and total effect of social capital on CHEI scores. With adjustment for covariates that predict diet quality, BOC and BRC had a medium size total effect on CHEI scores (*β =* 0.37 and 0.38, respectively). The impact of social capital on CHEI was mediated by both SS and NK. For BOC, the indirect effect via SS was greater than that via NK (0.12 and 0.05, respectively). Conversely, for BRC, the indirect effect via NK was greater than that via SS (0.10 and 0.05, respectively).

**Table 4:** Direct, indirect and total effect of social capital on CHEI.

	BOC			BRC		
	β	95% CI	% effect	β	95% CI	%
Direct	0.20	(0.06, 0.34)	54.1	0.23	(0.11, 0.36)	60.5
Indirect via SS	0.12	(0.05, 0.22)	32.4	0.05	(0.01, 0.10)	13.2
Indirect via NK	0.05	(0.02, 0.09)	13.5	0.10	(0.06, 0.15)	26.3
Total	0.37	(0.26, 0.49)	100.0	0.38	(0.27, 0.49)	100.0

β, standardized path coefficients; CI, confidence interval.

## Discussion

Our study revealed that the mean scores of CHEI among the two ethnic minority groups were 57.4, which is close to the result of a national-wide study conducted in 2011 (52.4) (33). These results indicate that the adherence to dietary guidelines remains low among the Chinese population, encompassing both the Han and minority. Consistent with prior research (34), our findings indicate that CHEI scores were lower in men and older individuals, highlighting the need to target these groups in future nutrition interventions. Notably, low adherence to dietary recommendations was observed in the consumption of dairy, beans, nuts, vegetables, fruits and animal-source foods. In fact, these foods, particularly beans, vegetables and fruits are fairly cheap and widely available in the areas under study. Likewise, there was no significant association between CHEI scores and income in the study. Over the past decades, the consumption of animal-source foods has continued to increase in Chinese adults while the consumption of dairy, vegetables and fruits remained at a low level (35). These findings suggest that factors beyond income significantly affect dietary intake. For instance, a study in Switzerland indicates that women who frequently dined with healthy eaters had higher diet quality and lower body mass index (36). Therefore, interpersonal relationships should be carefully considered in nutrition research.

Social capital positively predicts diet quality of the study population, both directly and indirectly. This finding corroborates the conclusion from previous studies that social capital is a protective factor of dietary guidelines adherence (37, 38). Additionally, a recent study conducted in Australia indicates that social isolation is correlated to poor diet quality in older adults (39). Findings of these studies provide evidence supporting the significance in enhancing social capital to promote healthy eating. A study in Nepal showed that incorporating nutrition training with community social capital development was associated with higher diet diversity and better child growth than isolated training programs alone (40). It is noteworthy that bonding and bridging social capital had nearly equal total effects on CHEI scores in this study. This finding is different from previous studies that emphasized the impact of bridging social capital (41). One possible explanation for the discrepancy could be attributed to the urban–rural dual structure in China, where rural residents typically have less bridging social capital than their urban counterparts (42). In this study, all subjects were from rural areas. Moreover, people usually eat with family members and friends. Hence, it is reasonable to anticipate that people within dinning networks would have a significant effect on individual’s dietary intake.

Nutrition knowledge and social support are crucial factors that influence individual dietary intakes. The Healthy China Action (2019–2030) has incorporated improving nutrition knowledge and creating supportive environments as key steps to achieve and maintain healthy eating among residents (43). However, compared to individuals from developed areas (27, 28), both nutrition knowledge and social support levels are lower in the study population. In our mediation analysis, we discovered that higher social capital was related to increased levels of nutrition knowledge and social support, which in turn were related to a higher CHIE score. Specifically, bonding capital was found to be more closely linked to social support, while bridging capital was more closely associated with nutrition knowledge. This finding is consistent with previous social capital and health research that people tend to receive support through homogeneous networks and novel information or ideas through heterogeneous networks (44, 45). This suggests the importance of considering the distinct roles of bonding and bridging capital when designing intervention programs. Additionally, a few studies have explored other pathways linking social capital to dietary intake. For example, a recent study in Lithuania showed that social capital could enhance a healthier diet among young adults by reducing psychological distress (46). Given the multifaceted nature of social capital, further research is needed to fully understand the relationships between social capital and dietary habits.

The study has several limitations that should be noted. First, due to cross-sectional study design, causal relations between dietary intake and related factors cannot be determined. Second, data for this study was specifically collected from two ethnic minority groups in southwest China. Therefore, caution is needed to generalize these findings to other populations. Third, we measured general social support instead of diet-specific social support, which may potentially influence the results (47). Last but not least, although our study showed good model fit, some important factors were not included in the model, such as food environment and self-efficacy (48, 49). Despite the limitations, the present study is one of the first to investigate the underlying mechanisms linking social capital to dietary guidelines adherence in Chinese context. Future studies are advised to explore additional factors to enhance understanding of this relationship. It is also recommended that future studies verify the findings of this study through longitudinal research involving diverse populations.

## Conclusion

Our study indicates that social capital may promote dietary guidelines adherence partly by improving nutrition knowledge and social support. This finding suggests a penitential avenue for advancing both the process and final ultimate goals of the Healthy China Action simultaneously. It is plausible to posit that nutrition promotion initiatives would be more successful if they actively integrate the development of social capital. Considering the complex relationship between social factors and dietary behaviors, further research is warranted, particularly in marginalized populations.

## Data availability statement

The original contributions presented in the study are included in the article/Supplementary material, further inquiries can be directed to the corresponding author.

## Ethics statement

The studies involving humans were approved by the Institutional Review Board at Yunnan Provincial Center for Disease Control and Prevention. The studies were conducted in accordance with the local legislation and institutional requirements. The participants provided their written informed consent to participate in this study.

## Author contributions

QZ: Conceptualization, Formal analysis, Writing – original draft. CH: Formal analysis, Writing – original draft. QW: Data curation, Investigation, Writing – review & editing. WS: Data curation, Investigation, Writing – review & editing. XZ: Data curation, Investigation, Writing – review & editing. BY: Writing – review & editing. XM: Writing – review & editing. ZL: Conceptualization, Writing – original draft.
