# Supplementary material for: Social capital and healthy eating among two ethnic minority groups in Yunnan Province, Southwest China: the mediating role of social support and nutrition knowledge
Source: Front Nutr. 2024 May 31;11:1273851. doi: 10.3389/fnut.2024.1273851 (PMC11176612; doi:10.3389/fnut.2024.1273851)
Supplement: Supplementary file 1 [file Data_Sheet_1.docx]

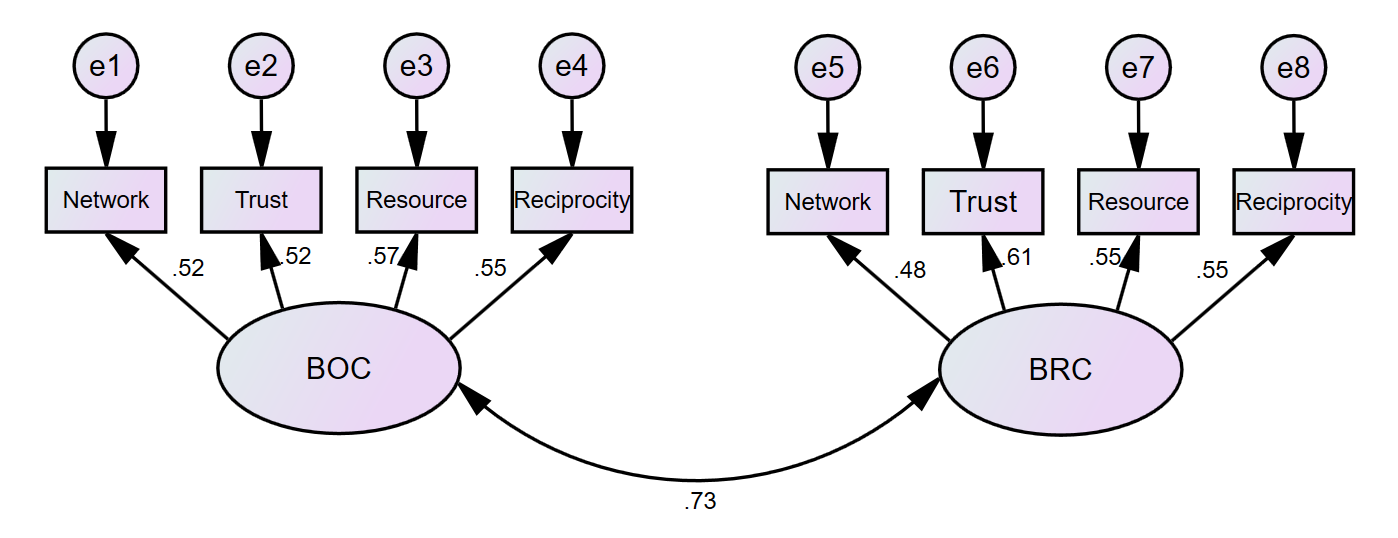


Supplementary Figure 1. Confirmation factor analyses of the PSCS-16

(*χ^2^/df*=1.81, *P*=0.02; CFI=0.96; RMSEA=0.03; TLI=0.98)


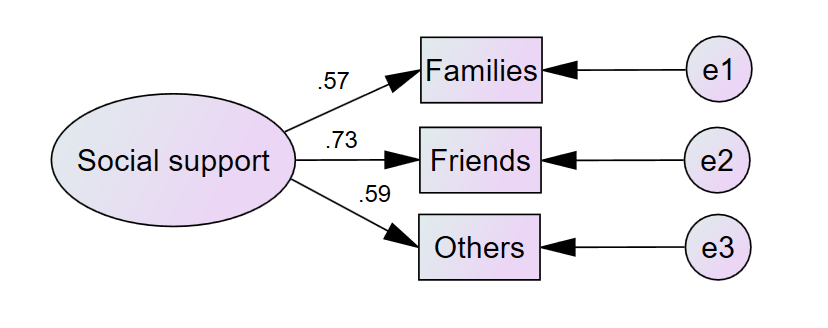


Supplementary Figure 2. Confirmation factor analyses of the MSPSS

(CFI=1.00, RMSEA=0.00)

Supplementary Table 1 CHEI components and standard for scoring


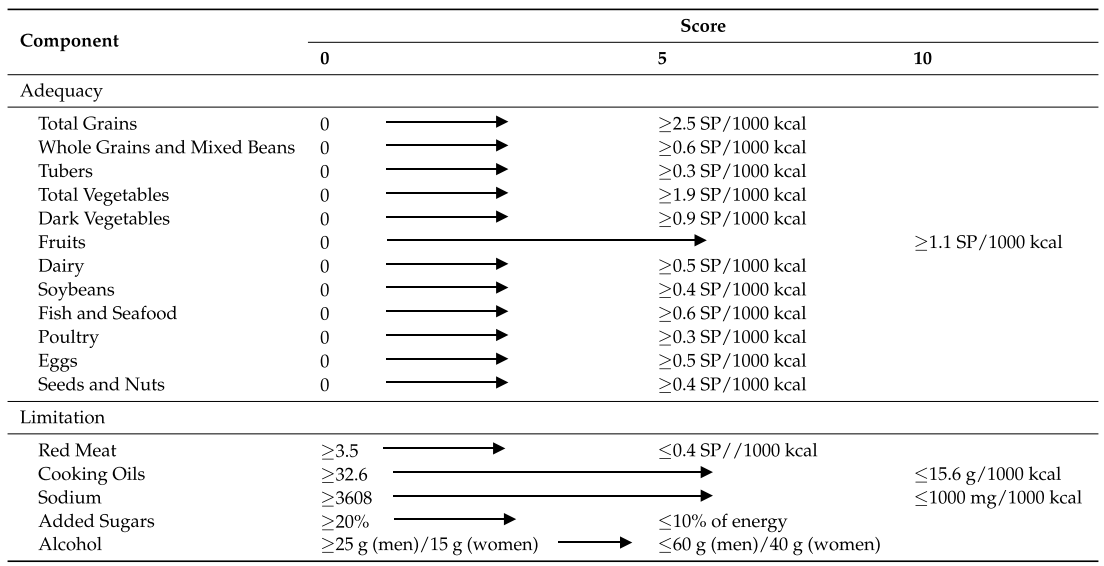


SP: Standard Portion.

Supplementary Table 2 Effect of social capital on the component scores of CHEI

| Scores of | BOC | | BRC | |
| --- | --- | --- | --- | --- |
|  | β | *P value* | β | *P value* |
| Grains and tubers | 0.09 | 0.13 | 0.08 | 0.13 |
| Vegetables and fruits | 0.10 | 0.20 | 0.37 | <0.01^**^ |
| Animal-source foods | 0.14 | 0.07 | 0.19 | 0.01^*^ |
| Dairy, beans and nuts | 0.23 | <0.01^**^ | 0.24 | <0.01^**^ |
| Cooking oils and salt | 0.35 | <0.01^**^ | 0.08 | 0.32 |

β: standardized path coefficients; ^*^ *P*<0.05, ^**^ *P*<0.01.
